# Transactions of the Ninth International Medical Congress

**Published:** 1889-05

**Authors:** John B. Hamilton


### Transactions of the Ninth International Medical Congress.

*Dear Sir*:—There are a number of volumes of the Transactions of the Ninth International Medical Congress on hand, belonging to members who have omitted to notify me of their change of address. On being notified of their present address, the volumes will be sent by express. Very truly yours,

JOHN B. HAMILTON.

